# Analysis of risk factors and prevention strategies of shoulder joint stiffness after traumatic proximal humeral fracture in patients with osteoporosis

**DOI:** 10.3389/fmed.2025.1641583

**Published:** 2026-02-06

**Authors:** Qiming Liu, Yue Qin, Hao Zhang, Weiwei Guo, Teng Ma, Min Zhang

**Affiliations:** 1 Department of Trauma and Orthopedics, General Hospital of Ningxia Medical University, Yinchuan, Ningxia, China; 2 Department of Cardiology, General Hospital of Ningxia Medical University, Yinchuan, Ningxia, China

**Keywords:** orthopedic, proximal humeral fractures, rehabilitation physiotherapy, risk factors, shoulder stiffness

## Abstract

**Objective:**

To explore the risk factors of shoulder stiffness after traumatic proximal humeral fracture in patients with osteoporosis, and to develop targeted prevention strategies to provide a basis for clinical optimization of perioperative management.

**Method:**

A retrospective analysis of 236 patients with osteoporosis and proximal humeral fractures treated from January to December 2024 was performed. According to whether shoulder stiffness occurred at 6 months after surgery, they were divided into stiffness group (119 cases) and non-stiffness group (117 cases). Clinical data such as age, body mass index (BMI), smoking history, and preoperative physical therapy were collected. Univariate and multivariate logistic regression analysis were used to analyze risk factors.

**Results:**

The incidence of shoulder stiffness was 50.4%. Univariate analysis showed that the stiffness group was older (52.83 ± 6.65 years vs. 43.31 ± 6.48 years), P24 kg/m^2^ ratio was higher (73.1% vs. 50.4%, *p* < 0.001), more smokers (42.9% vs. 19.7%, *p* < 0.001), and lower preoperative physical therapy rate (10.9% vs. 25.6%, *p* = 0.003). Multivariate analysis confirmed that advanced age (OR = 1.297), overweight (OR = 5.599), smoking (OR = 3.270) and prolonged course of disease (OR = 2.409) were independent risk factors, while preoperative standardized physical therapy was a protective factor (OR = 0.187). Subgroup analysis further indicated no significant difference in the incidence of shoulder stiffness between plate and nail fixation methods. ROC curve analysis demonstrated that age and course of disease possessed high predictive value (AUC = 0.857 and 0.770, respectively), whereas the predictive value of a history of standard physical therapy was relatively low (AUC = 0.570).

**Conclusion:**

Patients with osteoporosis undergoing surgery for proximal humeral fractures may develop shoulder joint stiffness. To optimize perioperative management, a three-tiered prevention strategy centered on smoking cessation, weight management and early physical therapy is recommended, supplemented by personalized comprehensive preventive measures.

## 1 Introduction

Osteoporosis (OP) is a systemic bone disease characterized by reduced bone mass and destruction of bone microstructure. Its core pathological manifestation is imbalance between bone resorption and bone formation, resulting in increased bone fragility and significantly increased fracture risk. The researches have indicated that, about 200 million people worldwide suffer from osteoporosis, of which the prevalence rate of women over 50 years old is as high as 30–50%, and that of men is about 15–22% (1, 2). Osteoporotic fractures have become a global public health problem. Especially in the elderly, a slight external force can lead to fractures of the hip, vertebral body and proximal humerus. Proximal Humeral Fracture (PHF) is one of the common types of fractures in patients with osteoporosis, accounting for 4–5% of systemic fractures. The incidence of PHF increases exponentially with age (3, 4). With the aggravation of social aging, the clinical treatment and postoperative rehabilitation of PHF have become increasingly prominent. Despite advances in surgical techniques, postoperative shoulder stiffness (Shoulder Stiffness) is still one of the major complications affecting the functional recovery of patients, with an incidence of up to 30–50%, which seriously reduces the quality of life and daily activities of patients (5).

Postoperative shoulder stiffness is mainly manifested as limited range of motion, pain and decreased function of the shoulder joint. Its mechanism is complex, involving multiple factors such as joint capsule contracture, soft tissue adhesion, muscle atrophy and abnormal neuromodulation (6). At present, domestic and foreign studies have focused on the effects of surgical methods (such as plate fixation, intramedullary nail or artificial joint replacement) on postoperative function, but there are few studies on the risk factors of postoperative stiffness in this special group of osteoporosis patients (7, 8). Patients with osteoporosis may further increase the risk of postoperative stiffness due to abnormal bone metabolism, delayed bone healing, and accelerated muscle atrophy (9). In addition, the role of preoperative rehabilitation interventions (such as physical therapy, early functional exercise) in preventing postoperative stiffness has not been fully explored.

In recent years, a number of studies have explored the related factors of stiffness after PHF. Robinson et al. found that advanced age, female, and fracture severity (Neer classification III-IV) were significantly associated with postoperative stiffness (10). Research indicates that shoulder stiffness following proximal humeral fractures typically manifests between 2 and 4 weeks post-injury (11), with the majority of patients experiencing significant functional improvement within 6 months of surgery. However, most of these studies did not specifically target patients with osteoporosis, and the analysis of controllable factors [such as preoperative rehabilitation, smoking, body mass index (BMI), etc.] was limited. Moreover, there is a lack of comprehensive analysis integrating both patient-specific characteristics and modifiable clinical factors to formulate stratified prevention strategies. In patients with osteoporosis, due to the decrease of bone quality and the decrease of microfracture repair ability, the postoperative rehabilitation process may be slower than that of ordinary patients. In addition, complications (such as diabetes, hypertension) may further affect postoperative recovery (12–15). Therefore, it is of great clinical significance to identify the independent risk factors of stiffness after PHF in patients with osteoporosis and to develop targeted prevention strategies.

The purpose of this study was to explore the risk factors of shoulder stiffness after traumatic PHF in patients with osteoporosis through retrospective analysis, focusing on age, BMI, smoking, preoperative rehabilitation intervention and other controllable factors, and to establish a multivariate logistic regression model to screen independent predictors. Based on the results of the study, individualized prevention strategies were proposed to optimize perioperative management, reduce the incidence of postoperative stiffness, and improve the level of functional recovery of patients. Perioperative rehabilitation protocols and the timing of surgery represent key factors that can be proactively managed. These interventions can significantly mitigate the potential adverse impact of patient-specific characteristics (uncontrollable factors) on surgical outcomes, thereby providing theoretical support for the clinical development of personalized interventions.

## 2 Materials and methods

### 2.1 Research subjects

In this study, a retrospective cohort study was conducted to select 236 patients with proximal humeral fractures and osteoporosis admitted to our hospital from January to December 2024 as the study subjects. All patients underwent open reduction and internal fixation (ORIF) and were followed up for more than 6 months. The patients were divided into stiffness group (119 cases) and non-stiffness group (117 cases) according to whether shoulder stiffness occurred at 6 months after operation (see section 2.2 for diagnostic criteria). The patient’s age, gender, BMI, smoking history, comorbidities (hypertension/diabetes), preoperative physical therapy (standardized physical therapy was defined as ≥3 times/week for more than 2 weeks), course of disease (time from injury to operation), etc. were recorded.

Inclusion criteria: (1) Age ≥ 40 years, in line with the WHO diagnostic criteria for osteoporosis (bone mineral density T value ≤ − 2.5); (2) Traumatic proximal humeral fractures (Neer type II-IV) were diagnosed by imaging; (3) ORIF surgery was performed, and internal fixation methods included locking plate or intramedullary nail; (4) The patients were followed up for more than 6 months, and the clinical data were complete. Exclusion criteria: (1) Pathological fracture (such as tumor, infection, etc.); (2) Rotator cuff injury, brachial plexus injury or preoperative limitation of shoulder joint activity were combined; (3) Serious heart, lung, liver, kidney dysfunction, can not tolerate surgery or rehabilitation training; (4) Postoperative loss of follow-up or lack of clinical data.

### 2.2 Diagnostic criteria for shoulder stiffness

Shoulder stiffness was defined as a significant limitation in passive range of motion (ROM) of the shoulder joint occurring after surgery, not attributable to other causes such as rotator cuff tear or nerve injury. This was operationalized based on previous literature and clinical standards (16) as meeting any of the following criteria: active flexion <120° (measured by protractor, patient sitting, scapula fixed); external rotation <30° (elbow flexion 90°, upper arm close to the trunk); internal rotation lower than the level of L3 vertebral body (assessed by the spinal segment accessible to the thumb).

### 2.3 Preoperative standard physical therapy protocol

The term “preoperative standard physical therapy” referred to a structured regimen initiated after diagnosis but before surgery. For the purpose of this study, it was defined as supervised physiotherapy sessions conducted at least 3 times per week for a minimum duration of 2 weeks. The protocol included: (1) Passive pendulum exercises to maintain joint mobility; (2) Codman’s exercises performed with therapist assistance to prevent capsular adhesion; (3) Isometric contractions of the shoulder girdle muscles (deltoid, supraspinatus) to maintain muscle tone without stressing the fracture site. Patients were instructed to avoid active movement of the fractured limb. Adherence was monitored through therapy session records.

### 2.4 Statistical analysis

Statistical analyses were performed using SPSS software (version 25.0). Descriptive statistics were presented as mean ± standard deviation (x̄ ± s) for continuous variables and as frequencies (n, %) for categorical variables. For univariate analysis, inter-group comparisons were conducted using the independent samples t-test for continuous data and the Chi-square (χ^2^) test for categorical data. Variables yielding a *p* value <0.05 in the univariate analysis were subsequently entered into a multivariate logistic regression model (Enter method) to identify independent risk factors for shoulder stiffness. Multicollinearity among the independent variables was assessed using the Variance Inflation Factor (VIF), with a VIF < 5 considered acceptable. The goodness-of-fit of the final logistic regression model was evaluated with the Hosmer-Lemeshow test, and its discriminatory power was quantified by the Area Under the Receiver Operating Characteristic (ROC) curve (AUC). A two-tailed *p*-value < 0.05 was considered statistically significant.

## 3 Results

### 3.1 Results of single factor analysis in patients with shoulder stiffness

**Figure 1:**
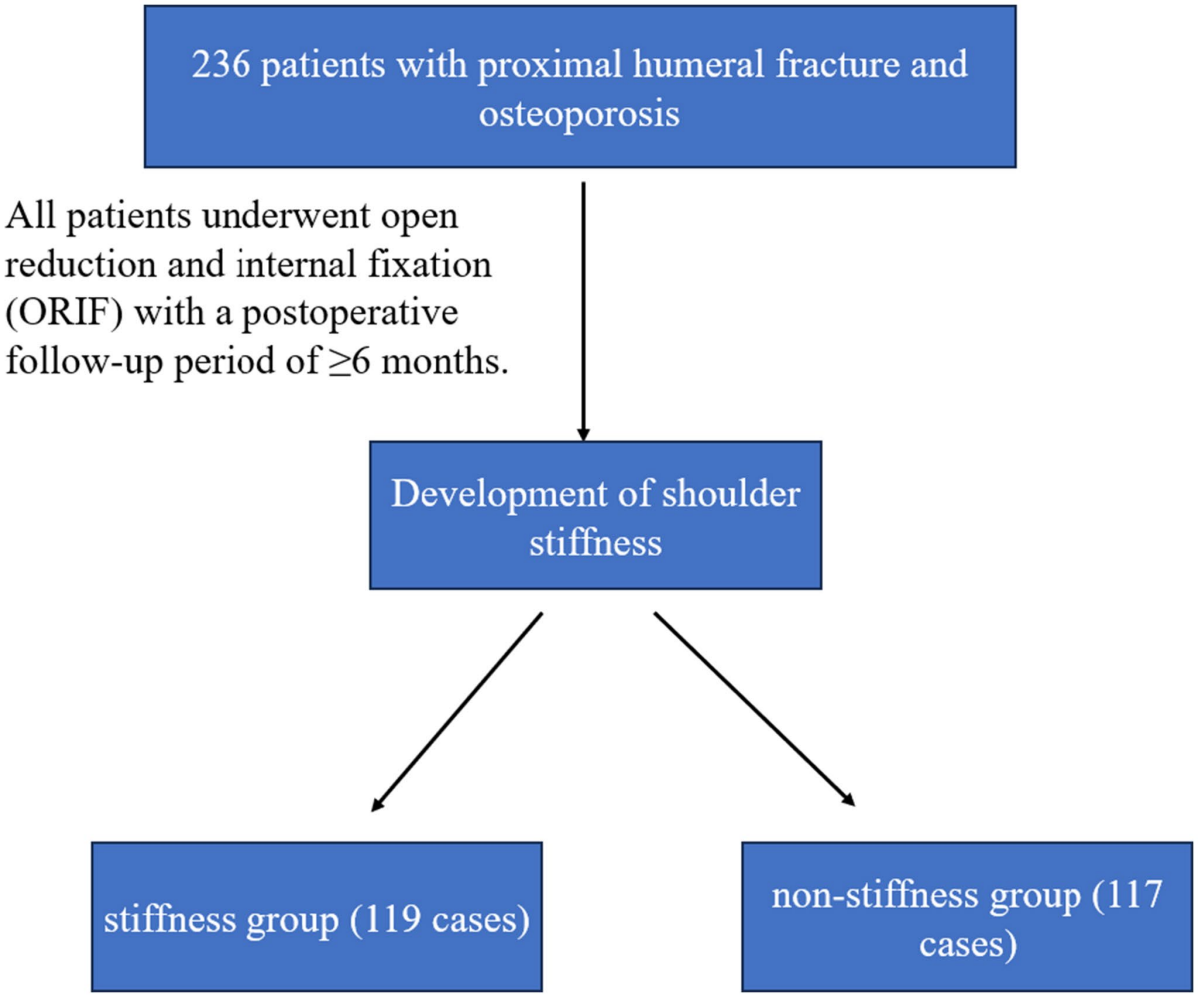
Patient screening, grouping, and follow-up flowchart.

A total of 236 patients with osteoporosis and proximal humeral fractures were included in this study. Among them, 119 (50.4%) had shoulder stiffness (stiffness group) and 117 (49.6%) had no stiffness (non-stiffness group). Univariate analysis showed that there were significant differences in multiple clinical features between the two groups (Table 1). The screening and grouping process for the study subjects is clearly illustrated in Figure 1.

**Table 1:** Single factor analysis results of shoulder joint stiffness patients.

Variable	Stiffness group (*n* = 119)	Non-stiffness group (*n* = 117)	*t*/*x*^2^	*P*
Age	52.83 ± 6.65	43.31 ± 6.48	11.132	<0.001
Gender			0.446	0.504
Male	56	50		
Female	63	67		
BMI index > 24 kg·m^2^/number	87	59	12.865	<0.001
Smoke/number	51	23	14.752	<0.001
Complicated with hypertension/number	46	52	0.814	0.367
Complicated with diabetes mellitus/number	43	45	0.137	0.712
Occupation type			0.052	0.820
Sedentary type	35	36		
Non-sedentary type	84	81		
Preoperative standard physical therapy			8.575	0.003
Yes	13	30		
No	106	87		
Injured side			1.424	0.233
Dominant side	83	73		
Non-dominant side	36	44		
Previous history of misdiagnosis/case	16	11	0.952	0.329
Course of Disease/month	8.45 ± 1.35	6.87 ± 1.58	8.291	<0.001

BMI, Body Mass Index. Data presented as mean ± standard deviation or number (n) and percentage (%).

In terms of demographic characteristics, the average age of patients in the stiffness group was significantly higher than that in the non-stiffness group (52.83 ± 6.65 years vs. 43.31 ± 6.48 years, *p* < 0.001), but there was no significant difference in gender distribution (*p* = 0.504) and affected side (dominant side/non-dominant side, *p* = 0.233) between the two groups. Body mass index analysis showed that patients with BMI > 24 kg / m^2^ had a significantly higher proportion in the stiffness group (73.1% vs. 50.4%, *p* < 0.001).

Among lifestyle factors, the proportion of smokers in the rigid group was significantly higher than that in the non-rigid group (42.9% vs. 19.7%, *p* < 0.001). In terms of clinical characteristics, there was no significant difference between the two groups in hypertension (*p* = 0.367), diabetes (*p* = 0.712) and work type (*p* = 0.820).

Analysis of treatment-related factors found that the proportion of patients receiving standardized preoperative physiotherapy was significantly higher in the non-rigid group (25.6% vs. 10.9%, *p* = 0.003). In addition, the time from injury to surgery in the rigid group was significantly longer than that in the non-rigid group (8.45 ± 1.35 months vs. 6.87 ± 1.58 months, *p* < 0.001).

### 3.2 Multivariate analysis of risk factors for shoulder stiffness

The patient’s stiffness was used as the dependent variable (0 = stiffness group, 1 = non-stiffness group), and the five variables in the univariate analysis were used as independent variables for multivariate analysis. The assignment of independent variables is shown in Table 2. Multivariate Logistic regression analysis (Table 3) identified 5 independent influencing factors. Age (OR = 1.297, 95% CI: 1.195–1.408, *p* < 0.001), BMI (OR = 5.599, 95% CI: 2.248–13.941, *p* < 0.001), smoking (OR = 3.270, 95% CI: 1.254–8.525, *p* = 0.015) and course of disease (OR = 2.409, 95% CI: 1.763–3.290, *p* < 0.001) were independent risk factors for shoulder stiffness. It was worth noting that preoperative standard physical therapy was a protective factor (OR = 0.187, 95% CI: 0.055–0.638, *p* = 0.007), which can reduce the risk of stiffness by 81.3%. The model goodness of fit test showed that the Hosmer-Lemeshow test *p* > 0.05, indicating that the model fitted well.

**Table 2:** Assignment table.

Factor	Assignment
Dependent variable	
Is the shoulder joint stiff	0 = Non-stiffness group; 1 = Stiffness group
Independent variable	
Age	Actual value
BMI index > 24 kg·m^2^	0 = Yes; 1 = No
Smoke	0 = Yes; 1 = No
Preoperative standard physical therapy	0 = Yes; 1 = No
Course of disease	Actual value

BMI, Body Mass Index.

**Table 3:** Multivariate analysis of risk factors for shoulder stiffness.

Factor	*β* value	*SE*	*Wald χ*^2^ value	*P*-value	*OR* value	95% confidence interval	
						Lower limit	Upper limit
Age	0.260	0.042	38.910	0.000	1.297	1.195	1.408
BMI	1.723	0.465	13.693	0.000	5.599	2.248	13.941
Smoke	1.185	0.489	5.873	0.015	3.270	1.254	8.525
Preoperative standard physical therapy	−1.676	0.626	7.169	0.007	0.187	0.055	0.638
Course of Disease	0.879	0.159	30.521	0.000	2.409	1.763	3.290

BMI, Body Mass Index.

### 3.3 Predictive value of various indicators for stiffness occurrence

**Figure 2:**
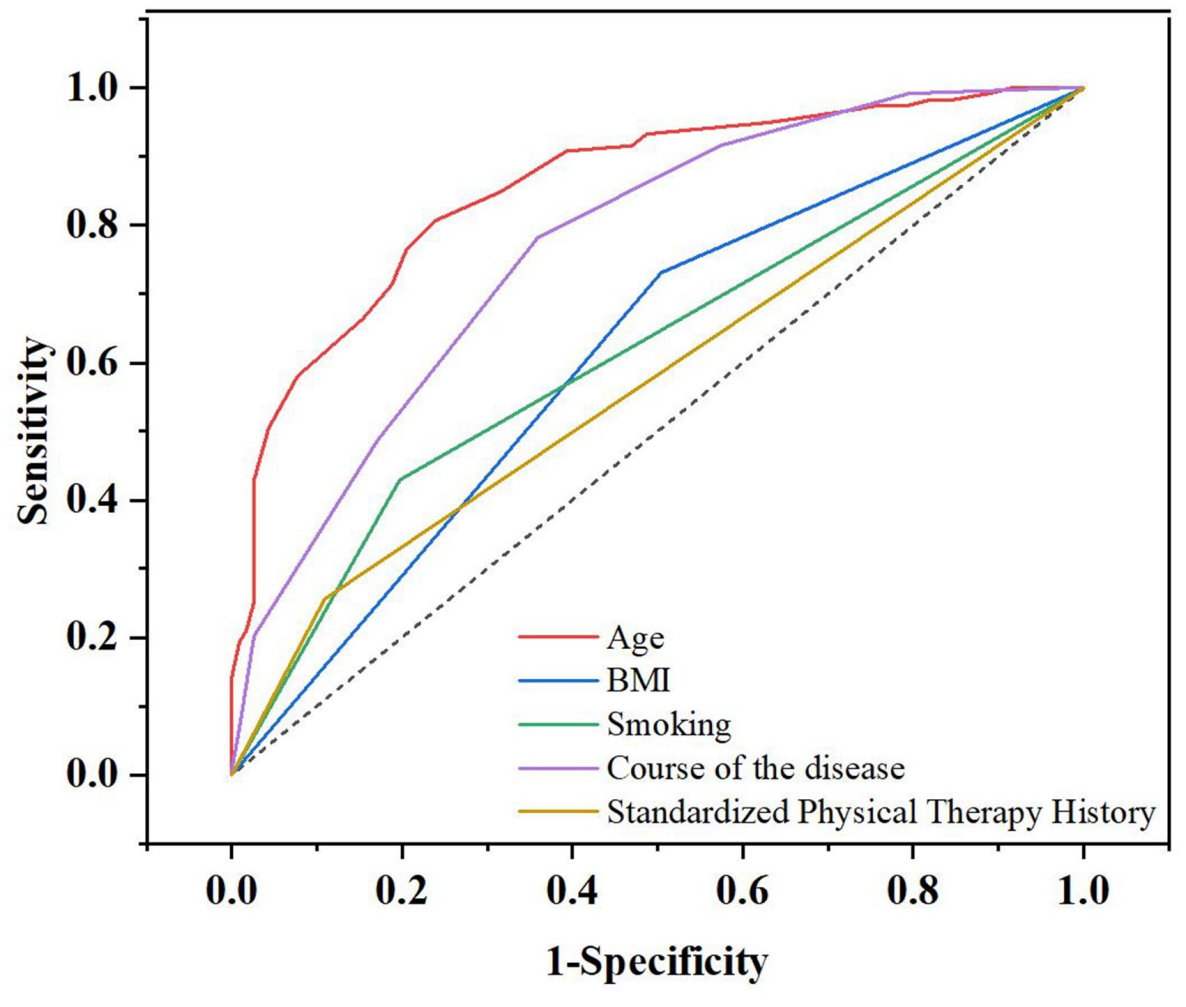
Receiver operating characteristic curve. BMI, body mass index.

To assess the predictive efficacy of various indicators, we constructed ROC curves (Figure 2). The analysis, summarized in Table 4, revealed that age demonstrated the highest predictive value (AUC = 0.857, 95% CI: 0.809–0.904), followed by the course of disease (AUC = 0.770, 95% CI: 0.710–0.829). In contrast, the predictive values of BMI, smoking history, and a history of standard physical therapy were relatively limited, with AUCs of 0.613, 0.616, and 0.570, respectively.

**Table 4:** Predictive value of various indicators for the occurrence of stiffness.

Factor	AUC	95%CI-Low	95%CI-Up	Sensitivity (%)	Specificity (%)
Age	0.857	0.809	0.904	80.7	76.1
BMI	0.613	0.541	0.685	73.1	49.6
Smoking	0.616	0.544	0.688	42.9	80.3
Course of disease	0.770	0.710	0.829	78.2	64.1
Preoperative standard physical therapy	0.570	0.501	0.647	25.6	89.1

BMI, Body Mass Index.

### 3.4 Subgroup analysis: comparison between plate and nail fixation

To further evaluate whether the type of internal fixation influenced the development of shoulder stiffness, we conducted a subgroup analysis comparing patients who underwent locking plate fixation versus those who received intramedullary nail fixation. Among the 236 patients, 158 (66.9%) were treated with locking plates and 78 (33.1%) with intramedullary nails. The baseline characteristics of the two fixation groups are summarized in Table 5. No significant differences were observed in age, gender, BMI, smoking status, preoperative physical therapy, or course of disease between the two fixation groups (all *p* > 0.05), indicating balanced baseline comparability.

**Table 5:** Baseline characteristics of patients stratified by fixation method.

Variable	Plate fixation (*n* = 158)	Nail fixation (*n* = 78)	*P*-value
Age (years)	48.2 ± 7.3	47.8 ± 7.1	0.674
Female, *n* (%)	85 (53.8%)	45 (57.7%)	0.564
BMI > 24 kg/m^2^, *n* (%)	98 (62.0%)	48 (61.5%)	0.941
Smokers, *n* (%)	45 (28.5%)	29 (37.2%)	0.165
Preoperative physical therapy, *n* (%)	32 (20.3%)	11 (14.1%)	0.243
Course of disease (months)	7.6 ± 1.5	7.8 ± 1.6	0.331
Neer classification, *n* (%)			0.420
– Type II	56 (35.4%)	32 (41.0%)	
– Type III	72 (45.6%)	34 (43.6%)	
– Type IV	30 (19.0%)	12 (15.4%)	
Shoulder stiffness, *n* (%)	81 (51.3%)	38 (48.7%)	0.712

Data are presented as mean ± standard deviation or number (percentage). BMI: Body Mass Index.

The incidence of shoulder stiffness did not differ significantly between the plate and nail groups (51.3% vs. 48.7%, *p* = 0.712). Within the stiffness group (n = 119), 78 patients (65.5%) had undergone plate fixation and 41 (34.5%) nail fixation, which was proportionate to the overall distribution of fixation methods. Multivariate logistic regression, adjusting for age, BMI, smoking, preoperative therapy, and course of disease, was performed to assess the independent effect of fixation type. As shown in Table 6, the type of fixation (nail vs. plate) was not an independent risk factor for shoulder stiffness (adjusted OR = 1.12, 95% CI: 0.63–1.98, *p* = 0.698).

**Table 6:** Multivariate logistic regression analysis for shoulder stiffness, incorporating fixation method.

Factor	Adjusted OR	95% confidence interval	*P*-value
Fixation method			
Nail (Ref: Plate)	1.12	0.63–1.98	0.698
Age (per year increase)	1.30	1.20–1.41	<0.001
BMI > 24 kg/m^2^	5.55	2.23–13.82	<0.001
Smoking	3.15	1.21–8.19	0.019
Preoperative physiotherapy	0.19	0.05–0.65	0.008
Course of disease (per month)	2.42	1.77–3.31	<0.001

OR: Odds Ratio; Ref: Reference category. The model confirms that after adjusting for significant covariates, the type of internal fixation is not an independent risk factor for postoperative shoulder stiffness.

## 4 Discussion

Clinical optimization is fundamentally a patient-risk-oriented, comprehensive and individualized management approach. Its core lies in systematically identifying modifiable risk factors and implementing targeted interventions at the appropriate juncture. This retrospective study identified several key modifiable and non-modifiable risk factors for shoulder stiffness following PHF surgery in osteoporotic patient. The results showed that the incidence of postoperative shoulder stiffness was as high as 50.4%, which was significantly higher than the 30–40% reported in the previous literature (8). This may be related to the special population of this study (osteoporosis patients) and the strict diagnostic criteria used. The following is an in-depth discussion of the main findings.

Age was proved to be an independent risk factor for shoulder stiffness (OR = 1.297). This result is consistent with the conclusion of Robinson et al. (17). The possible mechanisms include: (1) the decrease of muscle mass and strength in elderly patients affects the postoperative rehabilitation effect; (2) Age-related increased tendency of joint capsule fibrosis; (3) Delayed fracture healing caused by osteoporosis. It was worth noting that this study found that the risk of stiffness in patients over 50 years old increased sharply, suggesting that more active preventive measures should be implemented for patients in this age group. Furthermore, this study found that, BMI > 24 kg/m^2^ emerged as the most influential risk factor for shoulder stiffness in this study (OR = 5.599). The association may be explained through several potential mechanisms. First, excessive body weight increases mechanical load on the glenohumeral joint, potentially compromising fracture stability and impairing soft tissue healing. Second, adipose tissue functions as an active endocrine organ, secreting proinflammatory cytokines such as IL-6 and TNF-*α*, which may exacerbate local joint inflammation and capsular fibrosis (18, 19). These findings highlight the clinical importance of preoperative weight management, particularly in patients with complex Neer type III–IV fractures, who are at higher risk of compromised outcomes. Although the association between high BMI and shoulder joint stiffness may theoretically be bidirectional—where prior shoulder pain limiting activity leads to weight gain—research data supports obesity as the primary causal pathway elevating the risk of stiffness (20, 21). Smoking was significantly associated with an increased risk of stiffness (OR = 3.270), and its mechanism may involve: (1) nicotine inhibits osteoblast activity and delays fracture healing (22); (2) carbon monoxide leads to tissue hypoxia, affecting soft tissue repair; (3) smokers have low postoperative pain threshold and poor early activity compliance. Interestingly, subgroup analysis showed that smoking had a greater impact on men, which may be related to the fact that male smokers usually smoke more and have longer years of smoking. Preoperative standard physical therapy showed a significant protective effect (OR = 0.187), which was consistent with the results of a recent meta-analysis (23). It is particularly noteworthy that physiotherapy has a more significant protective effect on non-smokers, suggesting that there may be interactions between different risk factors. Age, BMI, smoking status, and preoperative rehabilitation interventions constitute patient-specific characteristics that are uncontrollable factors. Conversely, perioperative rehabilitation protocols and the timing of surgery represent key variables that can be actively modulated. These factors can significantly mitigate the potential adverse impact of patient-specific characteristics (uncontrollable factors) on surgical outcomes.

Prolonged course of disease (OR = 2.409) should be paid special attention as a controllable factor. In this study, the average preoperative waiting time of the stiff group was 1.58 months longer than that of the non-rigid group. The possible reasons include: (1) delayed diagnosis (the misdiagnosis rate of the stiff group was higher, although it was not statistically significant); (2) Poor control of complications delayed the timing of surgery; (3) Patients ‘fear of surgery (24). It is recommended to implement the ‘fracture green channel ‘for eligible patients, and the preoperative waiting time should be controlled within 2 weeks. Our study specifically investigated the role of comorbidities such as hypertension and diabetes, but no significant association was found in the univariate analysis. Therefore, they were not included in the final multivariate model. However, their potential role as confounders in the recovery process cannot be entirely ruled out and should be explored in larger studies.

Our subgroup analysis revealed no statistically significant difference in the incidence of shoulder stiffness between patients managed with locking plates versus intramedullary nails, even after adjusting for known risk factors. This suggests that, within the context of osteoporotic proximal humeral fractures treated with ORIF, the choice of implant may not be a primary determinant of postoperative stiffness. Both methods provide stable fixation, allowing for early rehabilitation, which appears to be more critical than the implant type itself. These findings align with recent meta-analyses indicating that functional outcomes following PHF are more strongly influenced by rehabilitation protocols and patient-specific factors than by the specific implant choice (25, 26). Nevertheless, surgeons should continue to select fixation methods based on fracture morphology, bone quality, and surgical expertise, with an emphasis on achieving stability that permits early motion.

This study innovatively found that there were interactions between different risk factors. For example, BMI > 24 kg/m^2^ has a greater impact on elderly patients, which may be related to the fact that elderly obese patients often have metabolic syndrome. In addition, the protective effect of preoperative physiotherapy is more significant in women, which may be related to women‘s better compliance with rehabilitation treatment. These findings provide a theoretical basis for individualized intervention. Based on the results of the study, we recommend three-level prevention strategies: (1) primary prevention (preoperative): smoking cessation intervention, weight management, preoperative physical therapy; (2) Secondary prevention (intraoperative): minimally invasive surgery, full joint capsule release; (3) Tertiary prevention (postoperative): rehabilitation training, multimodal analgesia and regular follow-up were started within 48 h.

The limitations of this study include: (1) the single-center retrospective design may affect the generalizability of findings; (2) the impact of bone metabolism indicators on stiffness was not assessed; (3) physiotherapy protocols were not entirely standardized; (4) propensity scoring or sensitivity analyses were not employed to adjust for baseline differences; (5) statistical power was insufficient due to sample size constraints, precluding subgroup analyses. Furthermore, the follow-up period was limited to 6 months, failing to encompass long-term functional outcomes and complications. Future prospective studies with larger sample sizes and extended follow-up periods are recommended to validate these findings and investigate differential effects across patient subgroups.

## 5 Conclusion

In summary, following proximal humeral fractures in patients with osteoporosis, postoperative shoulder stiffness occurs at a relatively high rate. Risk factors include advanced age (≥50 years), overweight/obesity (BMI > 24 kg/m^2^), smoking, and prolonged preoperative waiting times, whereas standard preoperative physiotherapy offers significant protective effects. The key findings of this study indicate that preoperative management should prioritize interventions targeting modifiable factors (smoking cessation, weight reduction, early physiotherapy) while enhancing perioperative management for elderly and overweight patients. Furthermore, establishing a fracture green channel to reduce preoperative waiting times is recommended.

## Data Availability

The original contributions presented in the study are included in the article/supplementary material, further inquiries can be directed to the corresponding author/s.
